# Considerations in Curriculum Design for Teaching About Race and Racism in Gerontology

**DOI:** 10.1093/geroni/igab046.474

**Published:** 2021-12-17

**Authors:** Darren Liu, Betty Burston

**Affiliations:** 1 Des Moines University, Des Moines, Iowa, United States; 2 University of Nevada, Las Vegas, Las Vegas, Nevada, United States

## Abstract

The recognition of structural racism as a public health crisis has enlarged the breadth and depth of discussions of the unique “Isms” in higher education classrooms. A significant number of undergraduate or graduate level gerontology programs participate in the delivery of training through instruction on the topics of aging, gerontology, or long-term care administration to future labor market participants. Absent from these classroom conversations, however, has been an analysis of how syntax, the analytical vocabulary, and other framing of such conversations can impact learners. While these educational programs provide highly critical information on disease, illness and injury prevention, self-care, population health, and other topics, professors can also introduce perspectives on race, racism, and how they may be related to the delivering of care to the aging population. Specifically, this session will introduce an example of curricular design for the identification of structural racism in the operation of long-term care institutions.

